# Brain size does not predict learning strategies in a serial reversal learning test

**DOI:** 10.1242/jeb.224741

**Published:** 2020-08-04

**Authors:** Annika Boussard, Séverine D. Buechel, Mirjam Amcoff, Alexander Kotrschal, Niclas Kolm

**Affiliations:** 1 Department of Zoology/Ethology, Stockholm University, Svante Arrhenius väg 18B, 10691 Stockholm, Sweden; 2 Behaviour Ecology, Wageningen University, De Elst 1, 6708wd Wageningen, The Netherlands

**Keywords:** Cognitive ability, Behavioural flexibility, Memory

## Abstract

Reversal learning assays are commonly used across a wide range of taxa to investigate associative learning and behavioural flexibility. In serial reversal learning, the reward contingency in a binary discrimination is reversed multiple times. Performance during serial reversal learning varies greatly at the interspecific level, as some animals adopt a rule-based strategy that enables them to switch quickly between reward contingencies. A larger relative brain size, generating enhanced learning ability and increased behavioural flexibility, has been proposed to be an important factor underlying this variation. Here, we experimentally tested this hypothesis at the intraspecific level. We used guppies (*Poecilia reticulata*) artificially selected for small and large relative brain size, with matching differences in neuron number, in a serial reversal learning assay. We tested 96 individuals over 10 serial reversals and found that learning performance and memory were predicted by brain size, whereas differences in efficient learning strategies were not. We conclude that variation in brain size and neuron number is important for variation in learning performance and memory, but these differences are not great enough to cause the larger differences in efficient learning strategies observed at higher taxonomic levels.

## Abstract (precis)

**Summary:** Relative brain size affects differences in advanced learning ability in small- and large-brained female guppies; however, regardless of brain size, they do not learn a generalized rule from earlier experience.

## INTRODUCTION

Cognitive ability varies greatly at all taxonomic levels (Shettleworth, 2010; Thornton and Lukas, 2012). More advanced cognitive abilities may enable an animal to use previous experience to develop efficient learning strategies (Hunter and Kamil, 1971; Mackintosh, 1974, 1988; Rumbaugh et al., 1996; Wilson et al., 1985). Here, we define an efficient learning strategy as the ability to generalize obtained information from earlier successful responses across situations by a learnt rule. By developing and adopting efficient learning strategies, an individual is able to switch faster between contingencies and solve novel problems than if restricted to, for instance, a fixed stimulus–response action pattern (Bitterman, 1965; Gonzalez et al., 1967). Differences in this aspect of cognition are well studied across a wide array of species. For example, macaws (*Diopsittaca nobilis*) outperform caiques (*Pionites melanocephala*) in both colour association and reversal learning tasks (van Horik and Emery, 2018), pinyon jays (*Gymnorhinus cyanocephalus*) solve spatial and visual serial discrimination problems faster than nutcrackers (*Nucifraga columbiana*) and scrub jays (*Aphelocoma californica*) (Bond et al., 2007), and bumblebees (*Bombus* spp.) outperform honeybees (*Apis* spp.) in odour discrimination problems (Sherry and Strang, 2015). The capacity to generalize information across situations and adopt an efficient learning strategy clearly differs between species. However, the proximate mechanisms causing this divergence remain unclear. Natural selection acts on the individual and the cause of individual variation and its potential consequences in cognitive evolution have been largely overlooked (Thornton and Lukas, 2012). To understand what causes the above-stated divergence in efficient learning strategies, it is important to also examine the proximate predictors of learning performance at the intraspecific level.

Reversal learning assays have been used in taxa ranging from insects to humans (Ashton et al., 2018; Bitterman, 1965; Bond et al., 2007; Buechel et al., 2018; Day et al., 1999; Izquierdo et al., 2016; Liu et al., 2016; Sherry and Strang, 2015). There are several strengths of reversal learning assays. First, reversal learning assays test several aspects of learning ability. Second, the neurological mechanisms underlying performance are comparable across multiple species. Third, the experimental protocol is largely standardized across different species. Initially, animals are trained in a binary discrimination task (e.g. visual, olfactory or spatial cues). After either a fixed number of trials or a pre-decided learning criterion, the reward contingency is reversed. In order to be rewarded, the animal thus has to inhibit the response towards the originally rewarded stimulus (A+) and switch to the previously unrewarded stimulus (B−). In this case A+ B− becomes A− B+. The switch in reward contingency is thought to be more cognitively demanding and involves different cognitive processes and brain regions from those for the initial discrimination task (Frank et al., 1972; Izquierdo et al., 2016; López et al., 2000; Watanabe, 2012; Buechel et al., 2018). The initial discrimination tests for associative learning ability, while the ability to reverse and learn the new reward contingency tests behavioural flexibility (Bitterman, 1965; Bond et al., 2007; Izquierdo et al., 2016; Shettleworth, 2010). In serial reversal learning, the reward contingency is reversed multiple times, which is considered to be even more demanding and specifically tests for differences in the formation of efficient learning strategies (Bitterman, 1965; Bond et al., 2007; Shettleworth, 2010). During serial reversals, some animals progressively improve their performance and make fewer errors as they continuously relearn the new reward contingency (Bitterman, 1965; Bond et al., 2007; Sherry and Strang, 2015). The ability to improve requires that the individual generalizes information based on earlier experience and adopts an efficient learning strategy to maximize the rewarded responses (Bitterman, 1965; Bond et al., 2007; Shettleworth, 2010).

In reversal learning assays, a striking variation in performance, post-reversal recovery rate and degree of efficient learning strategies is found, both across and within species (Ashton et al., 2018; Bitterman, 1965; Bond et al., 2007; Buechel et al., 2018; Day et al., 1999; Elias, 1970; Lucon-Xiccato and Bisazza, 2014; Sherry and Strang, 2015; van Horik and Emery, 2018). In serial reversal assays, some species perform very well in serial reversal learning, whereas others fail to both relearn and progressively improve over serial reversals (Bitterman, 1965; Lucon-Xiccato and Bisazza, 2014; Patrick et al., 1967; Warren, 1960). One key component that has been put forward to predict performance in serial reversal learning is relative brain size (Bitterman, 1965; Buechel et al., 2018; van Horik and Emery, 2018; Elias, 1970). This idea is partially supported at the intraspecific level, where artificially selected large-brained guppies and mice outperformed their small-brained conspecifics (Buechel et al., 2018; Elias, 1970). However, serial reversal learning may be considered cognitively more demanding than reversal learning as, in order to progressively improve over serial reversals, at least one additional cognitive process is required, i.e. the ability to generalize information by a learnt rule. To date, it is still unknown whether intraspecific variation in relative brain size also causes variation in performance and efficient learning strategies during serial reversal learning.

To test the hypothesis that relative brain size predicts the ability to adopt an efficient learning strategy, we used female guppies, *Poecilia reticulata* W. Peters 1859, artificially selected for small and large relative brain size and with known differences in neuron numbers (Marhounová et al., 2019), in a serial reversal learning assay. Previous experiments with brain size-selected lines have shown that large-brained guppies outperform small-brained guppies in a number of cognitively demanding tasks (Herczeg et al., 2019). These include associative numerical learning (Kotrschal et al., 2013a), spatial cognition (Kotrschal et al., 2015a) and reversal learning (Buechel et al., 2018). The apparent enhanced cognitive abilities of large-brained guppies have also been shown to be advantageous in ecologically relevant situations such as mate choice assessment (Bloch et al., 2018; Corral-Lopez et al., 2017), predator inspection behaviour (van der Bijl et al., 2015) and survival under predator threat (Kotrschal et al., 2015b). We quantified individual performance in binary colour discrimination over 10 serial reversals. As the ability to generalize previously gained information and adopt an efficient learning strategy is cognitively demanding, we hypothesized that a larger relative brain size will generate cognitive advantages facilitating performance in this test. We thus expected large-brained females to make fewer errors and adopt an efficient learning strategy that would result in a faster reversal learning rate over serial reversals.

## MATERIALS AND METHODS

### Brain size-selected guppies

The experiment was performed in accordance with ethical applications approved by the Stockholm Animal Research Ethical Permit Board (Dnr: N173/13 and 223/15). We used *n*=96, 7–8 month old female offspring from 7th generation guppies artificially selected for relative brain size, i.e. brain mass relative to body length (Kotrschal et al., 2013a), with associated differences in neuron number (Marhounová et al., 2019). In the 5th generation, the artificial selection had resulted in 15.4% difference in relative brain size and 11.9% difference in neuron number (Marhounová et al., 2019). Briefly, descendants of wild-caught guppies from high-predation areas in the Quare River, Republic of Trinidad and Tobago, were used to set up three independent breeding stocks (replicates). From each of these, one up- and one down-selected line were created, resulting in six brain size selection lines; 75 breeding pairs per replicate formed the parental strains. For details on the artificial selection, see Kotrschal and colleagues (2013a). Only females were used in this experiment as males have been difficult to motivate with a food reward (Fuss and Witte, 2019; Kotrschal et al., 2013b). Fish were kept with constant aeration, in 25±1°C water temperature, on a 12 h:12 h dark:light cycle. Fish were fed 6 days per week with flake food and live *Artemia nauplii*.

### Experimental apparatus

**Fig. 1.**
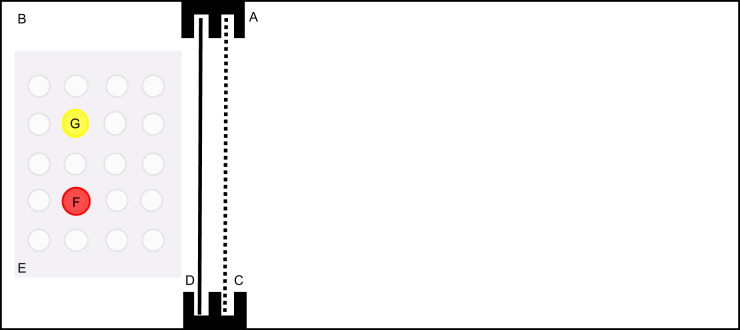
**Schematic diagram of the serial reversal learning set-up.** The tank consisted of a home compartment (A) and a conditioning chamber (B). These were separated by a transparent sliding door (C) and an opaque sliding door (D). All training took place in the conditioning chamber. A white plate (E) with 20 holes (10 mm in diameter, 5 mm deep) was placed at the bottom in the conditioning chamber. Animals were trained to discriminate between a red plastic disc (F) and a yellow plastic disc (G) and find an *Artemia* underneath the rewarded stimulus.

We used the experimental set-up described by Buechel and colleagues (2018) and first used by Lucon-Xiccato and Bisazza (2014). The 7 l experimental tanks were divided into a home compartment and a conditioning chamber (Fig. 1). To avoid unnecessary handling stress, individuals were held in the experimental tanks for the duration of the experiment. The fish were physically isolated but visual contact was allowed between adjacent home compartments to avoid negative isolation effects on learning (Bouton, 2007; Petrazzini et al., 2012). However, visual contact between adjacent conditioning chambers was prevented to avoid social learning effects (Brown and Laland, 2002; Laland and Williams, 1997; Reader et al., 2003). All training took place in the conditioning chamber. The home compartment and the conditioning chamber were divided by an opaque and a transparent sliding door. The opaque doors prevented the females from perceiving any visual cues from the arrangement by the experimenter. The transparent doors allowed the females to habituate to and assess the arrangement before entering the conditioning chamber. The trial started with the opening of the opaque door; 10 s later, we opened the transparent door. To prevent observer bias, the experiment was conducted blind and experimental tanks were only identified by running numbers.

### Pre-training

First, the females were trained to dislodge a black plastic disc (14 mm in diameter) placed on a white plate with 20 equispaced holes (10 mm in diameter, 5 mm deep) to access one frozen adult *Artemia* underneath. During the first trials, the disc only partially covered the well. Over successive trials, we increased well coverage until it was completely covered. All females could easily dislodge the disc after 30 trials. This set-up is ecologically relevant as it makes use of guppies' natural behaviour to forage underneath leaves and other plant parts (Houde, 1997).

### Colour discrimination learning

We trained 96 females (48 each of small and large brained) to discriminate between one red and one yellow disc and to associate one of the stimuli with the food reward. Half of all small- and large-brained females were trained to associate the red stimulus with a food reward, whereas the other half of all small- and large-brained females were trained to associate the yellow stimulus with the food reward. We chose stimuli colour in consideration of mate and food choice preferences of female guppies. Female guppies are known to prefer orange colours as they signal high quality in both mates and food (Houde, 1997; Rodd et al., 2002). The disc with the rewarded conditioned stimulus (CS+) was moveable, whereas the unrewarded conditioned stimulus (CS−) was unmovable because of a glued-on knob fixed in the well. In order to control for olfactory cues, food was placed underneath both the rewarded and the unrewarded disc. As mentioned above, the CS+ colour was counterbalanced and randomly distributed across the brain size-selected lines and replicates to control for any innate preference and colour bias. The females were given six trials per day over five consecutive days (i.e. 30 trials), with a 2 day pause prior to each reversal. To prevent side bias, the position (left- or right-hand side) of the CS+ was randomly chosen for each trial, with no more than two consecutive trials in the same position. For each trial, we scored the first push on either of the discs as either correct or incorrect. If a female did not push any of the discs within 120 s, that trial was counted as a no-choice trial. The time limit was chosen based on our experience in training guppies in this set-up (Buechel et al., 2018) and for logistic reasons such that relevant information was collected while permitting a relatively large sample size. For incorrect and no-choice trials, we gave each female 15 min to make a correct choice before we moved the rewarded disc 5 mm to the side to allow easy access to the food. This ensured that all females received the same number of positively reinforced trials throughout the experiment. During training, all females were tested in a randomized order, with trials typically running between 08:15 h and 17:00 h.

### Serial reversal learning assay

Following the initial colour discrimination training, the reward contingency was reversed, i.e. CS+ became CS− and vice versa, for 10 serial reversals. The trial procedure and duration were identical to the procedure described in the colour discrimination learning. We established a fixed number of trials (30) per reversal with reference to the previously established fast colour discrimination ability in female guppies (Buechel et al., 2018; Lucon-Xiccato and Bisazza, 2014).

### Data analysis

We performed all statistical analyses in R statistical software (version 3.5.1, http://www.R-project.org/), with the *glmer* function in *lme4* packages, version 1.1-18-1 used for mixed modelling (http://lme4.r-forge.r-project.org). In order to determine how relative brain size might explain variation in learning ability, we used generalized linear mixed models (GLMMs) with binomial error distributions and logit link functions (0=incorrect response, 1=correct response). We analysed the initial colour discrimination task and the serial reversal tasks separately, as the initial colour discrimination task tests associative learning ability, whereas serial reversal learning also tests behavioural flexibility and efficient learning strategies. Continuous variables were always centred at midpoint prior to analyses. Brain size was structured as a two-level categorical factor (small and large). Initially, all models included brain size nested in replicate as a random effect, but replicate returned a zero variance that caused singular fit. To control for a potential effect of replicate we thus included it as a fixed effect in all models, but dropped it from the models as it was not significant (*P*>0.3) and inclusion of replicate did not improve model fit (decreased Akaike information criterion, AIC). Non-significant interactions (*P*>0.1) were excluded until the lowest AIC was met. Statistical significance was obtained by using the ANOVA function, specifying Type III Wald χ^2^ tests, in the *car* package (Fox and Weisberg, 2019).

The full model testing initial colour discrimination included the fixed effects rewarded colour, the interaction between brain size and trial, as well as random slope for fish ID [glmer syntax final model: success∼brain size+trial+rewarded colour+(trial|fish ID)].

The full model testing the hypothesis that reversal learning rate is predicted by relative brain size initially included trial number as a fixed effect. Trial caused scaling problems and was therefore dropped from the model and replaced by session number (one to five). Each session included six trials for each reversal. The full model included the fixed effects rewarded colour, and the interactions between brain size and session, and brain size and reversal as well as random slope for fish ID [*glmer* syntax final model: success∼brain size×session+reversal+rewarded colour+(reversal|fish ID)].

The last trial of each reversal was followed by a 2 day pause prior to the next reversal, as described above, which constitutes an opportunity to measure explicit long-term memory (Bailey et al., 1996). By comparing performance on the first trial of each reversal, we could thus test for differences in long-term memory. The full model testing the performance in the first trial per reversal included the fixed effects rewarded colour, the interaction between brain size and reversal, as well as random intercept for fish ID [*glmer* syntax final model: success∼brain size+reversal+rewarded colour+(1|fish ID)]. To test whether differences in performance in the first trial of each reversal were caused by differences in memory retrieval, we fitted an additional GLMM that compared the performance on the fifth day of each reversal between small- and large-brained females. The full model included the fixed effects rewarded colour, brain size and reversal, as well as random intercept for fish ID [*glmer* syntax full model: success∼brain size+reversal+rewarded colour+(1|fish ID)].

## RESULTS

### Colour discrimination learning

In the colour discrimination part of the experiment, animals learnt to associate a colour stimulus with a food reward (trial: χ^2^_1_=63.22, *P*<0.001). However, matching previous results, neither learning rate (slope of the learning curve) nor performance was predicted by brain size. During the last day of colour discrimination training, small- and large-brained females reached similar performance levels, with a raw data mean±s.e. of 93±3.7% correct responses for small-brained females versus 98±2.0% correct responses for large-brained females. We found no main effect of brain size (χ^2^_1_=0.20, *P*=0.65). Females trained on the red stimulus learnt to associate the stimulus colour with the reward at a faster rate than females trained on the yellow stimulus (χ^2^_1_=19.35, *P*<0.001); note, that rewarded stimulus colour was counterbalanced between brain size and replicates (see Materials and Methods).

### Reversal learning rate and performance across serial reversals

**Fig. 2.**
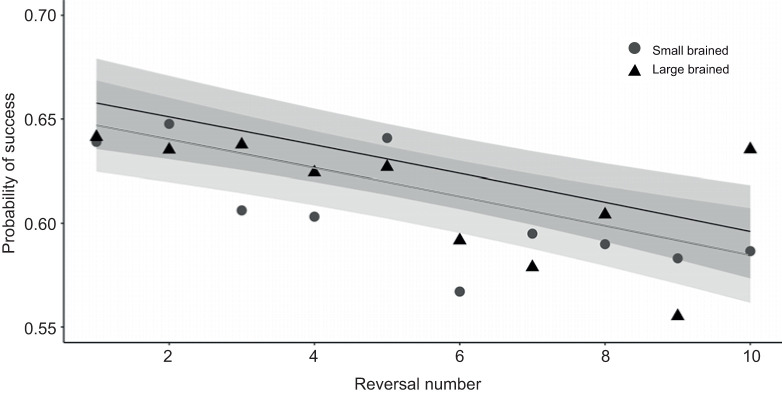
**Performance in serial reversal** **learning.** Performance and learning rate were measured as the proportion of correct responses in each trial across 10 serial reversals (see Materials and Methods). We found no evidence for the hypothesis that relative brain size predicts the ability to progressively improve performance over serial reversals and thereby develop an efficient learning strategy (brain size×reversal; χ^2^_1_=0.18, *P*=0.68). Raw mean data (based on 26,763 observations in total) for 48 small-brained and 48 large-brained female guppies across 10 serial reversals. The logistic regression slope estimates for small-brained (grey line) and large-brained (black line) females and 95% confidence interval (shading) are predictions obtained from a GLMM with binominal error distribution.

The brain size×reversal interaction was not significant (χ^2^_1_=0.18, *P*=0.68; Fig. 2), and was therefore excluded (as mentioned in Materials and Methods). This means that there was no evidence for differences in the ability to progressively improve and thereby adopt an efficient learning strategy between small- and large-brained females. For both brain size-selected lines, learning rate and performance during each reversal was negatively correlated with increasing number of reversals; error rate increased over serial reversals (χ^2^_1_=17.91, *P*<0.001). Performance on the last day of the final reversal was slightly above chance with a raw data mean±s.e. of 62.9±0.08% for small-brained versus 68.3±0.07% for large-brained females. Across reversals, the interaction between brain size and session number was significant (χ^2^_1_=6.44, *P*=0.01), suggesting a steeper average learning curve in large-brained compared with small-brained females within a week-long reversal. It was easier to switch from the rewarded red to the rewarded yellow stimulus than vice versa across reversals (χ^2^_1_=301.96, *P*<0.001). The model also revealed a significant main effect for session, but not for brain size (session: χ^2^_1_=791.99, *P*<0.001; brain size: χ^2^_1_=0.83, *P*=0.36).

### Explicit long-term memory

**Fig. 3.**
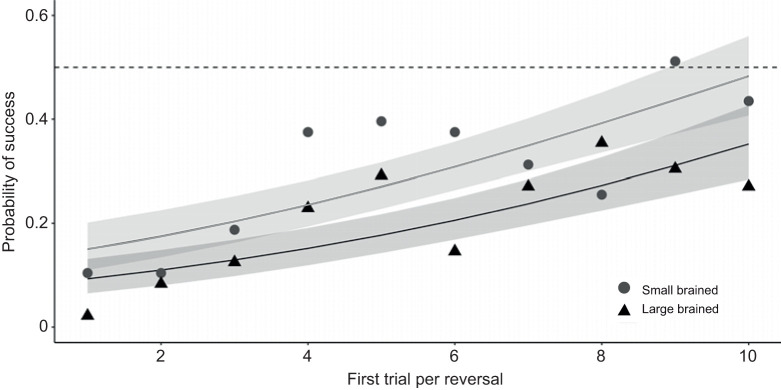
**Explicit long-term memory.** Memory was measured as the proportion of correct responses on the first trial of each reversal (preceded by a 2 day pause). We found that relative brain size predicted long-term memory, as large-brained females made more errors on the first trial of each reversal (brain size; χ^2^_1_=10.99, *P*<0.001). Raw mean data (based on 948 observations in total) and the logistic regression slope estimates for 48 small-brained and 48 large-brained female guppies in a serial reversal learning assay. The dotted horizontal line represents the 50% performance level. The logistic regression slope estimates for small-brained (grey line) and large-brained (black line) females and 95% confidence interval (shading) are predictions obtained from a GLMM with binominal error distribution.

On the first trial in nine out of the 10 reversals, large-brained females made significantly more errors and therefore performed at a lower level than small-brained females (χ^2^_1_=10.99, *P*<0.001; Fig. 3), suggesting an enhanced explicit long-term memory in large-brained females. Importantly, the performance on the last day of training for each reversal did not differ between the brain size-selected lines (χ^2^_1_=0.14, *P*=0.71), which indicates that the memory of what was learnt in the previous reversal was better stored in large-brained compared with small-brained females. The model for the last day of each reversal also revealed significant main effects for reversal and colour (reversal: χ^2^_1_=57.64, *P*<0.001; colour: χ^2^_1_=68.40, *P*<0001). For both brain size-selected lines, performance on the first trial of each reversal increased with increasing reversals (χ^2^_1_=42.66, *P*<0.001). Note that an increase in performance in the first trial of each reversal was an effect of increasingly lower performance levels at the end of the previous reversal. Memory retrieval was affected by rewarded stimulus colour (χ^2^_1_=14.73, *P*<0.001), suggesting that across reversals, the yellow stimulus was better stored in the long-term memory.

## DISCUSSION

We investigated the performance of small- and large-brained female guppies in a serial reversal learning assay. We found that large-brained females learnt the new reward contingency at a faster rate and also stored and retrieved information more efficiently in their explicit long-term memory as compared with their small-brained conspecifics. However, none of the brain size-selected lines progressively improved their performance over serial reversals. Rewarded colour was a strong predictor of performance and learning rate for both brain size-selected lines.

Differences in relative brain size, with associated differences in neuron number, affected variation in two distinct aspects of cognition in our experiment, as relative brain size predicted associative learning rate over serial reversals and predicted explicit long-term memory storage. However, learning rate during the initial discrimination did not differ significantly between the brain size-selected lines, something that has been observed in earlier assays on these selection lines (Buechel et al., 2018). Performance on the last day of training across serial reversals did not differ between the brain size-selected lines, whereas performance on the first trial of each reversal did. Large-brained females made more errors on the first trial across serial reversals, yet they reached the same performance level as small-brained females on the last day of each reversal. From this, we conclude that associative learning rate was faster in large-brained than in small-brained females in the more cognitively demanding reversal learning context; the significant interaction between brain size and session number corroborates this argument. Previous experimental studies have also found positive relationships between learning rate in demanding contexts and relative brain size, both in the brain size-selected guppies (Buechel et al., 2018) and in mice artificially selected for increased brain mass (Elias, 1970). The lower performance in the first trial of each reversal is most likely explained by differences in memory capacity. What is learnt towards the end of each reversal seems to be better stored in the explicit long-term memory of large-brained females. Phylogenetic comparative studies have revealed a correlation between whole-brain size and different aspects of memory storage, for instance between bird species (Garamszegi and Eens, 2004). Taken together, our results are mainly consistent with previous findings at the interspecific level in that relative brain size is advantageous in particularly demanding cognitive contexts (Benson-Amram et al., 2016; MacLean et al., 2014; Boire et al., 2002; Reader and Laland, 2002), whereas more fundamental aspects of cognition are probably less related to relative brain size (Buechel et al., 2018). However, we found no support for the proposal that relative brain size predicts the ability to learn from previous experience at the within-species level, as we found no difference in the ability to adopt an efficient learning strategy. The learning rate in both small-brained and large-brained females was instead strongly impaired over an increasing number of reversals, which almost led to extinction in the last reversals. Female guppies of the brain size-selected lines showed neither signs of stability in what was learnt during early reversals nor any flexibility to make new associations when contingencies were reversed repeatedly. We conclude that female guppies only rely on associative learning processes and apparently lack the ability to take advantage of previously successful discrimination in order to adopt an efficient learning strategy.

There are at least two possible explanations for why learning rate was impaired by serial reversals in both of the brain size-selected lines. First, ecological requirements create species-specific challenges that generate divergence in cognitive abilities (Shettleworth, 2010). Continuous changes in feeding opportunities or social complexity are ultimately thought to generate a flexible behaviour repertoire (Bond et al., 2007; Day et al., 1999; Sherry and Strang, 2015). In accordance with the existence of trade-offs between cognitive abilities, as proposed by Tellos-Ramos and colleagues (2019), the ecology of the guppy might favour spatial memory rather than the advanced learning abilities investigated here. Second, cognitive processes involved in the ability to adopt an efficient learning strategy are controlled by a specific region in the telencephalon (Frank et al., 1972; Izquierdo et al., 2016; López et al., 2000; Watanabe, 2012). Primates have among the largest relative telencephalon sizes in the animal kingdom (Finlay and Darlington, 1995), and telencephalon neuron number and density in parrots and many songbirds are equivalent to those of primates (Olkowitcz et al., 2016). Interestingly, species from these taxa are also known to typically perform well in serial reversal assays (Cauchoix et al., 2017; Boogert et al., 2011; Gosette, 1968; Gosette et al., 1966; van Horik and Emery, 2018). Brain region size typically varies between but not within species (Finlay and Darlington, 1995; Gould et al., 2013; Healy and Krebs, 1992; Lucas et al., 2004). Neither neuron density nor relative (to the rest of the brain) telencephalon volume differ between the brain size-selected lines (Kotrschal et al., 2017; Marhounová et al., 2019), which might explain why we found quantitative (i.e. a slightly higher learning rate) but not qualitative (i.e. negative effect of increasing reversals in both brain size-selected lines) differences in learning ability between the brain size-selected lines. We speculate that more advanced learning abilities are explained by the evolution of increased telencephalon size and/or neuron number, rather than by relative brain size and total number of neurons. Alternatively, individual variation in relative brain size and neuron number is too small for the detection of differences in advanced learning abilities in these selection lines.

The positive effect on discrimination learning rate with increasing number of reversals is generally substantially smaller in fish (Engelhardt et al., 1973; Fuss and Witte, 2019; Levin and Vergara, 1987; Lucon-Xiccato and Bisazza, 2014; Mackintosh and Cauty, 1971; Squier, 1969; Wodinsky and Bitterman, 1957) than in other vertebrates (Bitterman, 1965; Bond et al., 2007; Mackintosh and Cauty, 1971). Although there are examples of a positive effect on discrimination learning rate across reversals (see for example Engelhardt et al., 1973; Mackintosh and Cauty, 1971; Wodinsky and Bitterman, 1957), the majority of studies in fish have failed to show any progressive improvement over serial reversals (Behrend et al., 1965; Behrend and Bitterman, 1967; Bitterman et al., 1958; Bitterman, 1965; Engelhardt et al., 1973; Fuss and Witte, 2019; Gonzalez et al., 1967; Levin et al., 1984; Lucon-Xiccato and Bisazza, 2014; Patrick et al., 1967; Portavella and Vargas, 2005; Warren, 1960). Our results are thus consistent with many previous serial reversal learning assays in other species of fish. Another aspect of the results found in fish in this context is that, even when they show improvement over increasing reversals, they rarely improve their performance over serial reversals beyond the level of performance in the initial discrimination. It can thus be questioned whether the fish tested really understand ‘the principle of reversal’ (*sensu* Shettleworth, 2010).

A surprising finding during this experiment was the strong effect of the rewarded stimulus. During the initial colour discrimination part, female guppies from both brain size-selected lines made more errors with the yellow compared with the red stimulus. Red is well known to be a signal of strong biological significance for female guppies as, under natural conditions, it predicts feeding opportunities and male quality (Houde, 1997). It is thus not surprising that we found a bias for red, something that has been shown before in a learning context (Buechel et al., 2018). Interestingly, across serial reversals, more errors were made with the red compared with the yellow stimulus. This suggests a shift in this pre-existing bias, induced by an increased number of reversals. One possible explanation for this shift might be that as red is an important stimulus for female guppies, not being rewarded for pushing the red disc might be perceived as a strong negative experience. Over time, this might decrease the willingness to push the red disc. A shift in the pre-existing bias for important stimuli has been shown to change after both positive and negative experience in butterflies (Westerman et al., 2012) and spiders (Hebets, 2003; Hebets and Vink, 2007; Svensson et al., 2010) in mating contexts. However, whether the shift from red to yellow bias in this experiment is an experimental artefact or whether it has an adaptive value should be investigated further.

Although the relative brain size was not measured directly in the fish used in this experiment, all of the previous assays of relative brain size in both earlier and the current generation of selection have shown substantial differences (typically 12–15% differences in later generations) in relative brain size (Kotrschal et al., 2013a,b, 2017), brain volume (Kotrschal et al., 2017; Marhounová et al., 2019) and neuron numbers (Marhounová et al., 2019) between these selection lines. We therefore assume that these differences remain in the fish used in the current study.

To conclude, it is increasingly clear that in demanding contexts, a relatively larger brain size provides individuals with an enhanced cognitive ability. However, differences in learning ability and behavioural flexibility between the brain size-selected lines were not large enough to enable detection of the differences found at higher taxonomic levels (Bitterman, 1965; Bond et al., 2007). We speculate that the mechanisms causing variation in the ability to adopt an efficient learning strategy require larger variation in brain size, neuron number or region structure size than the differences in relative brain size observed in these selection lines. Additionally, based on both the results of the present study and those of previous experimental studies (Buechel et al., 2018; Elias, 1970), we hypothesize that while a relatively larger brain increases performance in species-specific tasks, it does not provide any additional learning ability. We thus propose that brain size-driven cognitive divergence within species is mostly quantitative in nature.

## References

[JEB224741C1] Ashton B. J., Ridley A. M., Edwards E. K. and Thornton A. (2018). Cognitive performance is linked to group size and affects fitness in Australian magpies. *Nature* 554, 364-367. 10.1038/nature25503 29414945 PMC5815499

[JEB224741C2] Bailey C. H., Bartsch S. and Kandel E. R. (1996). Toward a molecular definition of long-term memory storage. *Pros. Natl. Acad. Sci. USA* 93, 13445-13452. 10.1073/pnas.93.24.13445 PMC33629 8942955

[JEB224741C3] Behrend E. R. and Bitterman M. E. (1967). Further experiments on habit reversal in the fish. *Psycho. Sci.* 8, 363-362. 10.3758/BF03332241

[JEB224741C4] Behrend E. R., Domesick V. B. and Bitterman M. E. (1965). Habit reversal in the fish. *J. Comp. Physiol. Psychol.* 63, 407-411. 10.1037/h0022566 5891344

[JEB224741C5] Benson-Amram S., Dantzer B., Stricker G., Swanson E. M. and Holekamp K. E. (2016). Brain size predicts problem-solving ability in mammalian carnivores. *Pros. Natl. Acad. Sci. USA* 113, 2532-2537. 10.1073/pnas.1505913113 PMC4780594 26811470

[JEB224741C6] Bitterman M. E. (1965). Phyletic differences in learning. *Am. Psych.* 20, 396-410. 10.1037/h0022328 14301944

[JEB224741C7] Bitterman M. E., Wodinsky J. and Candland D. K. (1958). Some comparative psychology. *Amer. J. Psychol.* 71, 94-110. 10.2307/1419199 13521022

[JEB224741C8] Bloch N. I., Corral-López A., Buechel S. D., Kotrschal A., Kolm N. and Mank J. E. (2018). Early neurogenomic response associated with variation in guppy female mate preference. *Nat. Ecol. Evol.* 2, 1772-1781. 10.1038/s41559-018-0682-4 30297748 PMC6349141

[JEB224741C9] Boire D., Nicolakakis N. and Lefebvre L. (2002). Tools and brains in birds. *Behaviour* 139, 939-973. 10.1163/156853902320387918

[JEB224741C10] Bond A. B., Kamil A. C. and Balda R. P. (2007). Serial reversal learning and the evolution of behavioral flexibility in three species of North American corvids (*Gymnorhinus cyanocephalus, Nucifraga Columbiana, Aphelocoma californica*). *J. Comp. Psychol.* 4, 372-379. 10.1037/0735-7036.121.4.372 18085920

[JEB224741C11] Boogert N. J., Anderson R. C., Peters S., Searcy W. A. and Nowicki S. (2011). Song repertoire size correlates with detour reaching, but not with other cognitive measures. *Anim. Behav.* 81, 1209-1216. 10.1016/j.anbehav.2011.03.004

[JEB224741C12] Boussard A., Buechel S., Amcoff A., Kotrschal A. and Kolm N. (2020). Data from: Brain size does not predict learning strategies in a serial reversal learning test. *Dryad Digital Repository*. 10.5061/dryad.5mkkwh72s PMC7413604 32561630

[JEB224741C13] Bouton M. E. (2007). *Learning and Behavior: a Contemporary Synthesis.* Sutherland, MA: Sinaur Associates.

[JEB224741C14] Brown C. and Laland K. N. (2002). Social learning of a novel avoidance task in the guppy: conformity and social release. *Anim. Behav.* 64, 41-47. 10.1006/anbe.2002.3021

[JEB224741C15] Buechel S. D., Boussard A., Kotrschal A., van der Bijl W. and Kolm N. (2018). Brain size affects performance in a reversal learning test. *Pro. R. Soc. B Biol. Sci.* 285, 20172031 10.1098/rspb.2017.2031 PMC5805926 29367391

[JEB224741C16] Cauchoix M., Hermer E., Chaine A. S. and Morand-Ferron J. (2017). Cognition in the field: comparison of reversal learning performance in captive and wild passerines. *Sci. Rep.* 7, 12945 10.1038/s41598-017-13179-5 29021558 PMC5636806

[JEB224741C17] Corral-Lopez A., Bloch N. I., Kotrschal A., van der Bijl W., Buechel S. D., Mank J. E. and Kolm N. (2017). Female brain size affects the assessment of male attractiveness during mate choice. *Sci. Adv.* 3, e1601990 10.1126/sciadv.1601990 28345039 PMC5362185

[JEB224741C18] Day L. B., Crews D. and Wilczynski W. (1999). Spatial and reversal learning in congeneric lizards with different foraging strategies. *Anim. Behav.* 57, 393-407. 10.1006/anbe.1998.1007 10049480

[JEB224741C19] Elias M. F. (1970). Spatial discrimination reversal learning for mice genetically selected for differing brain size: a supplementary report. *Percept. Mot. Skills* 30, 239-245. 10.2466/pms.1970.30.1.239 5476110

[JEB224741C20] Engelhardt F., Woodard W. T. and Bitterman M. E. (1973). Discrimination reversal in the goldfish as a function of training conditions. *J. Comp. Physiol. Psychol.* 85, 144-150. 10.1037/h0034879

[JEB224741C21] Finlay B. and Darlington R. B. (1995). Linked regularities in the development and evolution of mammalian brains. *Science* 268, 1578-1584. 10.1126/science.7777856 7777856

[JEB224741C22] Fox J. and Weisberg S. (2019). *An R Companion to Applied Regression*, 3rd edn Thousand Oaks, CA: Sage.

[JEB224741C23] Frank A. H., Flood N. B. and Overmier J. B. (1972). Reversal learning in forebrain ablated and olfactory tract sectioned teleost *Carassius auratus**.* *Psychon Sci.* 26, 149-151. 10.3758/BF03335463

[JEB224741C24] Fuss T. and Witte K. (2019). Sex differences in color discrimination and serial reversal learning in mollies and guppies. *Curr. Zool.* 65, 323-332. 10.1093/cz/zoz029 31263491 PMC6595423

[JEB224741C25] Garamszegi L. Z. and Eens M. (2004). The evolution of hippocampus volume and brain size in relation to food hoarding in birds. *Ecol. Lett.* 7, 1216-1224. 10.1111/j.1461-0248.2004.00685.x

[JEB224741C26] Gonzalez R. C., Behrend E. R. and Bitterman M. E. (1967). Reversal learning and forgetting in bird and fish. *Science* 158, 519-521. 10.1126/science.158.3800.519 6069100

[JEB224741C27] Gosette R. L. (1968). Examination of retention decrement explanation of comparative successive discrimination reversal learning by birds and mammals. *Percept. Mot. Skills* 27, 1147-1152. 10.2466/pms.1968.27.3f.1147

[JEB224741C28] Gosette R. L., Frome Gosette M. and Riddell W. (1966). Comparisons of successive discrimination reversal performances among closely and remotely related avian species. *Anim. Behav.* 14, 560-564. 10.1016/S0003-3472(66)80060-X 5972814

[JEB224741C29] Gould K. L., Gilbertson K. E., Seyfer A. L., Brantner R. M., Hrvol A. J., Kamil A. C. and Nelson J. C. (2013). Differences in relative hippocampus volume and number of hippocampus neurons among five corvid species. *Brain Behav. Evol.* 81, 56-70. 10.1159/000345560 23364270 PMC4961358

[JEB224741C30] Healy S. D. and Krebs J. R. (1992). Food storing and the hippocampus in corvids: amount and volume are correlated. *Pro. R. Soc. B* 248, 241-245. 10.1098/rspb.1992.0068

[JEB224741C31] Hebets E. A. (2003). Subadult experience influences mate choice in an arthropod: exposed female wolf spiders prefer males of a familiar phenotype. *Pros. Natl. Acad. Sci. USA* 100, 13390-13395. 10.1073/pnas.2333262100 PMC263824 14597702

[JEB224741C32] Hebets E. A. and Vink C. J. (2007). Experience leads to preference: experienced females prefer brush-legged males in a population of syntopic wolf spiders. *Behav. Ecol.* 18, 1010-1020. 10.1093/beheco/arm070

[JEB224741C33] Herczeg G., Urszán T. J., Orf S., Nagy G., Kotrschal A. and Kolm N. (2019). Brain size predicts behavioural plasticity in guppies (*Poecilia reticulata*): an experiment. *J. Evol. Biol.* 32, 218-226. 10.1111/jeb.13405 30474900

[JEB224741C34] Houde A. E. (1997). *Sex, Color, and Mate Choice in Guppies.* Princeton, NJ: Princeton University Press.

[JEB224741C35] Hunter M. W. and Kamil A. C. (1971). Object-discrimination learning set and hypothesis behavior in the northern bluejay (*Cynaocitta cristata*). *Psychon. Sci.* 22, 271-273. 10.3758/BF03335950

[JEB224741C36] Izquierdo A., Brigman J. L., Radke A. K., Rudebeck P. H. and Holmes A. (2016). The neural basis of reversal learning: an updated perspective. *Neuroscience* 345, 12-26. 10.1016/j.neuroscience.2016.03.021 26979052 PMC5018909

[JEB224741C37] Kotrschal A., Rogell B., Bundsen A., Svensson B., Zajitschek S., Brännström I., Immler S., Maklakov A. A. and Kolm N. (2013a). Artificial selection on relative brain size in the guppy reveals costs and benefits of evolving a larger brain. *Curr. Biol.* 23, 168-171. 10.1016/j.cub.2012.11.058 23290552 PMC3566478

[JEB224741C38] Kotrschal A., Rogell B., Bundsen A., Svensson B., Zajitschek S., Brännström I., Immler S., Maklakov A. A. and Kolm N. (2013b). The benefit of evolving a larger brain: big-brained guppies perform better in a cognitive task. *Anim. Behav.* 86, e4-e6. 10.1016/j.anbehav.2013.07.011 24109149 PMC3791419

[JEB224741C39] Kotrschal A., Corral-Lopez A., Amcoff M. and Kolm N. (2015a). A larger brain confers a benefit in a spatial mate search learning task in male guppies. *Behav. Ecol.* 26, 527-532. 10.1093/beheco/aru227 25825587 PMC4374130

[JEB224741C40] Kotrschal A., Buechel S. D., Zala S. M., Corral-Lopez A., Penn D. J. and Kolm N. (2015b). Brain size affects female but male survival under predation threat. *Ecol. Lett.* 18, 646-652. 10.1111/ele.12441 25960088 PMC4676298

[JEB224741C41] Kotrschal A., Zeng H.-L., van der Bijl W., Öhman-Mägi C., Korschal K., Pelckmans K. and Kolm N. (2017). Evolution of brain region volumes during artificial selection for relative brain size. *Evolution* 71, 2942-2951. 10.1111/evo.13373 28986929

[JEB224741C42] Laland K. N. and Williams K. (1997). Shoaling generates social learning of foraging information in guppies. *Anim. Behav.* 53, 1161-1169. 10.1006/anbe.1996.0318 9236013

[JEB224741C43] Levin L. E. and Vergara E. (1987). Reversal learning in groups of the schooling fish *Aphyocharax erithrurus* on an avoidance paddle. *J. Comp. Psychol.* 101, 317-321. 10.1037/0735-7036.101.4.317

[JEB224741C44] Levin L. E., Vergara E. and Perez G. A. (1984). Aprendizaje en reversiones sucesivas de una discriminación simultánea en peces (*Aphyocharax* sp.) en paleta de evitación [Succesive reversal learning of a spatial simultaneous discrimination in fish (*Aphyocharax* sp.)]. *Acta Científica Venezolana* 35, 288-292.

[JEB224741C45] Liu Y., Day B. L., Summers K. and Burmeister S. S. (2016). Learning to learn: advanced behavioural flexibility in a poison frog. *Anim. Behav.* 111, 167-172. 10.1016/j.anbehav.2015.10.018

[JEB224741C46] López J. C., Broglio C., Rodríguez F., Thinus-Blanc C. and Sales C. (2000). Reversal learning deficit in a spatial task but not in a cued one after telencephalic ablation in goldfish. *Behav. Brain Res.* 109, 91-98. 10.1016/S0166-4328(99)00167-9 10699661

[JEB224741C47] Lucas J. R., Brodin A., de Kort S. R. and Clayton N. S. (2004). Does hippocampal size correlate with the degree of caching specialization? *Pros. R. Soc. B Biol. Sci.* 271, 2423-2429. 10.1098/rspb.2004.2912 PMC1523289 15590591

[JEB224741C48] Lucon-Xiccato T. and Bisazza A. (2014). Discrimination reversal learning reveals greater female behavioural flexibility in guppies. *Biol. Lett.* 10, 20140206 10.1098/rsbl.2014.0206

[JEB224741C49] Mackintosh N. J. (1974). *The Psychology of Animal Learning.* New York: Academic Press.

[JEB224741C50] Mackintosh N. J. (1988). Approaches to the study of animal intelligence. *Br. J. Psychol.* 79, 509-525. 10.1111/j.2044-8295.1988.tb02749.x

[JEB224741C51] Mackintosh N. J. and Cauty A. (1971). Spatial reversal learning in rats, pigeons, and goldfish. *Psychon. Sci.* 22, 281-282. 10.3758/BF03335956

[JEB224741C52] MacLean E. L., Hare B., Nunn C. L., Addessi E., Amici F., Anderson R. C., Aureli F., Baker J. M., Bania A. E., Barnard A. M. et al. (2014). The evolution of self-control. *Pros. Natl. Acad. Sci. USA* 20, 2140-2148. 10.1073/pnas.1323533111

[JEB224741C53] Marhounová L., Kotrschal A., Kverková K., Kolm N. and Nêmec P. (2019). Artificial selection on brain size leads to matching changes in overall number of neurons. *Evolution* 73, 2003-2012. 10.1111/evo.13805 31339177 PMC6772110

[JEB224741C54] Olkowitcz S., Kocourek M., Lučan R. K., Porteš M., Fitch W. T., Herculano-Houzel S. and Nemec P. (2016). Birds have primate-like number of neurons in the forebrain. *Pros. Natl. Acad. Sci. USA* 113, 7255-7260. 10.1073/pnas.1517131113 PMC4932926 27298365

[JEB224741C55] Patrick J., Capretta J. and Rea R. (1967). Discrimination reversal learning in the crayfish. *Anim. Behav.* 15, 6-7. 10.1016/S0003-3472(67)80003-4 6031112

[JEB224741C56] Petrazzini M. E. M., Agrillo C., Piffer L., Dadda M. and Bisazza A. (2012). Development and application of a new method to investigate cognition in newborn guppies. *Behav. Brain. Res.* 233, 443-449. 10.1016/j.bbr.2012.05.044 22677276

[JEB224741C57] Portavella M. and Vargas J. P. (2005). Emotional and spatial learning in goldfish is dependent on different telencephalic pallial systems. *Euro. J. Neruosci.* 21, 2800-2806. 10.1111/j.1460-9568.2005.04114.x 15926927

[JEB224741C58] Reader S. M. and Laland K. N. (2002). Social intelligence, innovation, and enhanced brain size in primates. *Pros. Natl. Acad. Sci. USA* 99, 4436-4441. 10.1073/pnas.062041299 PMC123666 11891325

[JEB224741C59] Reader S. M., Kendal J. R. and Laland K. N. (2003). Social learning of foraging sites and escape routes in wild Trinidadian guppies. *Anim. Behav.* 66, 729-739. 10.1006/anbe.2003.2252

[JEB224741C60] Rodd F. H., Hughes K. A., Grether G. F. and Baril C. T. (2002). A possible non-sexual origin of mate preference: are male guppies mimicking fruit? *Proc. R. Soc. Lond. B* 269, 475-481. 10.1098/rspb.2001.1891 PMC1690917 11886639

[JEB224741C61] Rumbaugh D. M., Savage-Rumbaugh E. S. and Washburn D. A. (1996). Toward a new outlook on primate learning and behaviour: complex learning and emergent processes in comparative perspective. *Jpn. Psychol. Res.* 38, 113-125. 10.1111/j.1468-5884.1996.tb00016.x 11541528

[JEB224741C62] Sherry D. F. and Strang C. G. (2015). Contrasting styles in cognition and behaviour in bumblebees and honeybees. *Behav. Processes* 117, 59-69. 10.1016/j.beproc.2014.09.005 25218105

[JEB224741C63] Shettleworth S. J. (2010). *Cognition, Evolution, and Behavior*. New York, NY: Oxford University Press.

[JEB224741C64] Squier L. H. (1969). Reversal learning improvement in the fish *Astronotus ocellatus* (Oscar). *Psychon. Sci.* 14, 143-144. 10.3758/BF03332753

[JEB224741C66] Svensson E. I., Eroukhmanoff F., Karlsson K., Runemark A. and Brodin A. (2010). A role for learning in population divergence of mate preferences. *Evolution* 64, 3101-3113. 10.1111/j.1558-5646.2010.01085.x 20629727

[JEB224741C67] Tellos-Ramos M. C., Branch C. L., Kozlovsky D. Y. and Pitera A. M. (2019). Spatial memory and cognitive flexibility trade-offs: to be or not to be flexible, that is the question. *Anim. Behav.* 147, 129-136. 10.1016/j.anbehav.2018.02.019

[JEB224741C68] Thornton A. and Lukas D. (2012). Individual variation in cognitive performance: developmental and evolutionary perspectives. *Phil. Trans. R. Soc. B* 367, 2773-2783. 10.1098/rstb.2012.0214 22927576 PMC3427550

[JEB224741C69] van der Bijl W., Thyselius M., Kotrschal A. and Kolm N. (2015). Brain size affects the behavioural response to predators in female guppies (*Poecilia reticulata*). *Pros. R. Soc. B.* 282, 20151132 10.1098/rspb.2015.1132 PMC4528528 26203003

[JEB224741C70] van Horik J. O. and Emery N. J. (2018). Serial reversal learning and cognitive flexibility in two species of Neotropical parrots (*Diopsittca nobilis* and *Pionites melanocephala*). *Behav. Processes* 157, 664-672. 10.1016/j.beproc.2018.04.002 29656091

[JEB224741C71] Warren J. M. (1960). Reversal learning by paradise fish (*Macropudus operucularis*). *J. Comp. Physiol. Psychol.* 53, 376-378. 10.1037/h0044187 13842934

[JEB224741C72] Watanabe S. (2012). *‘What’ and ‘Where’ Analysis and Flexibility in Avian Visual Cognition.* Oxford, UK: Oxford University Press.

[JEB224741C73] Westerman E. L., Hodgins-Davis A., Dinwiddie A. and Monteiro A. (2012). Biased learning affects mate choice in a butterfly. *Pros. Natl. Acad. Sci. USA* 109, 10948-10953. 10.1073/pnas.1118378109 PMC3390860 22689980

[JEB224741C74] Wilson B., Mackintosh N. J. and Boakes R. A. (1985). Transfer of relational rules in matching and oddity learning by pigeons and corvids. *Q. J. Exp. Psychol. Sect. B* 37, 313-332. 10.1080/14640748508401173

[JEB224741C75] Wodinsky J. and Bitterman M. E. (1957). Discrimination-reversal in the fish. *Amer. J. Pscychol.* 70, 569-567. 10.2307/1419447 13487826

